# Treatment of echinococcosis: albendazole and mebendazole – what else?[Fn FN1]

**DOI:** 10.1051/parasite/2014073

**Published:** 2014-12-22

**Authors:** Andrew Hemphill, Britta Stadelmann, Reto Rufener, Markus Spiliotis, Ghalia Boubaker, Joachim Müller, Norbert Müller, Daniela Gorgas, Bruno Gottstein

**Affiliations:** 1 Vetsuisse Faculty, Institute of Parasitology, University of Berne Länggass-Strasse 122 3012 Berne Switzerland; 2 Department of Clinical Veterinary Medicine, Clinical Radiology Bremgartenstrasse 109a 3012 Berne Switzerland

**Keywords:** Alveolar echinococcosis (AE), *Echinococcus multilocularis* chemotherapy, *In vitro* culture, Drugs, Host-parasite interaction

## Abstract

The search for novel therapeutic options to cure alveolar echinococcosis (AE), due to the metacestode of *Echinococcus multilocularis*, is ongoing, and these developments could also have a profound impact on the treatment of cystic echinococcosis (CE), caused by the closely related *Echinococcus granulosus s.l.* Several options are being explored. A viable strategy for the identification of novel chemotherapeutically valuable compounds includes whole-organism drug screening, employing large-scale *in vitro* metacestode cultures and, upon identification of promising compounds, verification of drug efficacy in small laboratory animals. Clearly, the current focus is targeted towards broad-spectrum anti-parasitic or anti-cancer drugs and compound classes that are already marketed, or that are in development for other applications. The availability of comprehensive *Echinococcus* genome information and gene expression data, as well as significant progress on the molecular level, has now opened the door for a more targeted drug discovery approach, which allows exploitation of defined pathways and enzymes that are essential for the parasite. In addition, current *in vitro* and *in vivo* models that are used to assess drug efficacy should be optimized and complemented by methods that give more detailed information on the host-parasite interactions that occur during drug treatments. The key to success is to identify, target and exploit those parasite molecules that orchestrate activities essential to parasite survival.

## Abstract (fr)

La recherche de nouvelles options thérapeutiques curatives de l’échinococcose alvéolaire (EA), due au métacestode d’*Echinococcus multilocularis*, est en progrès, et ses développements pourraient aussi avoir un profond impact sur le traitement de l’échinococcose kystique (EK), due au cestode très proche *Echinococcus granulosus s.l.* Plusieurs options sont explorées. Une stratégie efficace pour l’identification de composés nouveaux à activité chimiothérapique est représentée par le criblage de médicaments sur le micro-organisme entier, utilisant des cultures à grande échelle de métacestodes *in vitro* et, après identification de composés d’intérêt, la vérification de leur activité chez des animaux de laboratoire. La recherche actuelle est clairement centrée sur les médicaments et les classes de substances à activité antiparasitaire et anti-cancéreuse à large spectre qui sont déjà sur le marché ou en cours de développement dans d’autres applications. La mise à disposition d’informations complètes sur le génome d’*Echinococcus* et sur l’expression des gènes ainsi que des progrès significatifs à l’échelle moléculaire ouvrent maintenant la porte vers une approche plus ciblée pour la découverte de nouveaux médicaments, en permettant l’exploitation de voies métaboliques et d’enzymes indispensables au parasite. De plus, les modèles actuels, *in vitro* et *in vivo*, actuellement utilisés pour confirmer l’efficacité d’un médicament, devraient être optimisés et complétés par des méthodes qui permettraient d’obtenir des informations plus détaillés sur les relations hôte-parasite qui surviennent au cours des traitements. La clé du succès est d’identifier, de cibler et d’exploiter les molécules parasitaires qui orchestrent des activités essentielles à la survie du parasite.

## 1. Introduction

The genus *Echinococcus* includes seven to nine described species or genotypes [37], of which *Echinococcus multilocularis* (the small fox tapeworm) is the most pathogenic, and causes alveolar echinococcosis (AE) in humans. *E. multilocularis* is largely restricted to the Northern hemisphere and highest prevalence rates occur in Central Asia, Russia, North-Western China, and parts of Europe and Japan. *Echinococcus granulosus* (the small dog tapeworm) causes cystic echinococcosis (CE), occurs globally and represents the most common species found in the Mediterranean area, Central Europe, South America, Africa and Central Asia. In addition, CE exists as an imported disease in Western Europe and the USA [7]. Both parasites cause life-threatening disorders of serious public health and economic concern worldwide [62]. For AE for instance, although a rare infection, the severity of the disease results in an estimated 600,000 disability-adjusted life years (DALYs), which renders the impact of AE comparable to tropical diseases such as leprosy, dengue and schistosomiasis [7]. CE, but to some extent also AE, affects predominantly resource-poor communities. For AE, present also in industrialized countries with high economic standards, the number of cases is underestimated by public health authorities in many countries. For instance, the incidence rate for Germany, 0.07/100,000 persons, is probably underestimated by a factor of 3–5 [17]. These factors contribute to the fact that the development of new drugs for echinococcosis has not been a major focus of the pharmaceutical industry. Both AE and CE are neglected diseases, and emergence (or re-emergence), especially in developing countries, is likely, with an increasing economic impact due to the need for livelong treatments [63].

Humans represent an aberrant intermediate host for these parasites. Infection is acquired through the accidental ingestion of parasite eggs, with serious health consequences for those individuals in whom the disease develops. Eggs contain the infectious larval oncosphere, which actively penetrates the intestinal lining, and migrates via blood and lymphatic vessels to the target sites, mostly the liver and lungs. There, these oncospheres develop into the disease-causing metacestodes. Within these metacestodes, protoscolex development takes place in most intermediate hosts. If this type of infected intermediate host is ingested by a suitable definitive host, the life cycle is concluded [13]. Protoscolex development in humans infected with *E. multiloculari*s has only rarely been described.

Metacestodes are fluid-filled vesicles that are separated into (i) an inner germinal layer representing the living and metabolically active parasite tissue, and (ii) an outer, acellular compartment known as the laminated layer, mediating the direct physical contact with host immune and non-immune cells. The distal part of the germinal layer, the tegument, is directly associated with the inner surface of the laminated layer. The tegument is characterized by microvilli-like extensions termed microtriches, which protrude well into the matrix of the laminated layer, and increase the resorbing surface of the parasite. In addition, the germinal layer contains highly differentiated cell types including connective tissue, muscle cells, and glycogen storage cells, as well as many undifferentiated cells [8]. The outer laminated layer is a carbohydrate-rich structure that is synthesized by the parasite and secreted into its peripheral entities, and which, in terms of thickness, is much more prominent in *E. granulosus s.l.* metacestodes [13]. In contrast to *E. granulosus s.l.* metacestodes, *E. multilocularis* metacestodes are not surrounded by a very prominent host-derived adventitial layer. Instead, the parasite larva represents a multivesicular organism that reproduces asexually, by exogenous formation and budding of daughter vesicles. This process is often referred to as “progressive tumour-like growth” [1], and leads to the formation of a large and heterogeneous parasitic mass consisting of mostly peripheral, actively proliferating sites, and in many cases, centrally located necrotic tissue. Release of germinal layer cells into the blood or lymphatic vessels may lead to metastasis formation in other organs [1, 8, 13, 33]. Spontaneous resolution of AE leading to calcified lesions is possible, but it is not known how common this is [1, 3, 8, 13, 33]. Mass screenings in endemic areas have revealed that the number of established AE infections in humans is far lower than the number of sero-positive humans who had been exposed to the eggs of the parasite, but who remained clinically healthy [19]. This indicated that in certain individuals, innate or acquired immunity contributes to the control of parasite development after infection. However, the major focus of this review will be on drugs for the treatment of *Echinococcus* infections, presenting progressively growing and thus fully active metacestodes.

## 2. Advantages and pitfalls of the current treatments of AE

Conventionally, treatment of echinococcosis relies on surgery and/or chemotherapy depending on different factors such as metacestode size and location, viability status, the interaction between the expanding parasite and the adjacent host tissue, bacterial and fungal infection, and, in the case of CE, potential complications related to cyst rupture and spillage of protoscoleces [4, 5, 20].

In CE, radical resection of the cyst mass represents the traditional treatment strategy and is, in many instances, accompanied by chemotherapy. In addition, PAIR (puncture, aspiration, injection, re-aspiration) is a technique introduced in the mid-1980s. PAIR has been used in 700 patients, with promising results [4, 5]. Indications and rules on how and when to apply PAIR have been elaborated by an appropriate expert group [5]. For inoperable cases, chemotherapy with the benzimidazoles albendazole, mebendazole and the heterocyclic pyrazinoisoquinoline derivative praziquantel remains the only options [6, 14, 65, 66]. Adverse reactions against benzimidazoles under long-term chemotherapy may occur, but can be avoided by constant monitoring of drug serum levels (not possible in all countries). Mebendazole and albendazole may induce embryotoxic or teratogenic effects [14]. Praziquantel, exhibiting high efficacy against protoscoleces and metacestodes in animal experiments [6, 65, 66], was proposed to be applied during the month prior to surgery alongside albendazole, since this increased the number of human patients with non-viable protoscoleces compared to therapy with albendazole alone.

For AE, the prognosis is to some extent different. If surgery is carried out, it is always accompanied by chemotherapy using benzimidazoles, which should be continued for at least 2 years thereafter, and monitoring of patients should be continued for 10 years. Inoperable AE cases must undergo long-term chemotherapy, often life-long, which is based on albendazole and/or mebendazole [40, 43]. Clinical studies have shown that benzimidazoles have significantly increased the 10-year survival rate of inoperable or non-radically operated AE patients from 6–25% to 80–83% [7]. Despite this, albendazole and mebendazole exhibit a parasitostatic rather than a parasitocidal effect. In addition, hepatotoxicity occurs in some patients, and they are left without any other option for treatment. Therefore, recurrence rates after interruption of therapy are relatively high, especially in those patients not followed up with appropriate prognostic tools [43, 60]. Thus, besides benzimidazoles, other treatment options for AE are needed to improve the clinical status of infected and diseased patients. Respective compounds should exhibit better selectivity, a significantly improved therapeutic window, and they should act as a parasiticidal rather than a parasitostatic.

## 3. Assessment of novel drugs and drug targets to be explored for the treatment of AE

A substantial number of drugs have been evaluated for their potential use against AE *in vitro* and, in many instances also *in vivo*, employing a whole-organism based approach [12, 13]. These include mostly broad-spectrum anti-infective drugs, and drugs that inhibit cellular proliferation such as anti-cancer compounds. Most reports, however, have been anecdotal, since only relatively small numbers of compounds could be evaluated at a given time, and large-scale screening efforts were not possible. This has changed. The development of large-scale *in vitro* techniques for the maintenance, propagation and axenic culture of *E. multilocularis* metacestodes [2, 53] has now paved the way for whole metacestode drug screening assays with a higher throughput. In addition, the current genomic sequencing efforts [64, 69] have opened new avenues, and conventional organism screening can now be complemented by more targeted molecular genetic approaches.

### 3.1. Effects of anti-infective agents on *E. multilocularis* metacestodes

Earlier animal experimentation studies in rodents demonstrated parasitostatic effects of mitomycin C, piperazine and quinolone derivatives, alkylaminoethers and propargylic alcohols, either at a lower level or comparable to benzimidazoles. Other drugs such as praziquantel or alpha-difluoromethylornithine were ineffective (reviewed in [12, 13, 48]). Nitazoxanide, a broad-spectrum anti-parasitic drug belonging to the thiazolide family, exhibited promising anti-echinococcal activities *in vitro* [28, 29]. The drug was also effective in experimentally infected mice, and an albendazole-nitazoxanide combination treatment was shown to be more effective than albendazole alone [58, 59]. However, the drug did not keep its promises: neither nitazoxanide monotherapy nor albendazole-nitazoxanide combination therapies were effective in human patients suffering from AE [21, 61]. However, a few studies suggested that nitazoxanide may be an effective treatment option in CE, particularly in patients with progressive disease who are receiving conventional therapy [38, 67, 68].

The *in vitro* efficacy of a series of compounds against *E. multilocularis* metacestodes, including albendazole, artemether, caspofungin, itraconazole, ivermectin, methiazole, miltefosine, nitazoxanide, rifampicin and trimethoprim/sulfamethoxazole, was investigated by Reuter et al. [44], and the authors found that albendazole, itraconazole, methiazole and nitazoxanide effectively destroyed parasite vesicles *in vitro* but effects were only parasitostatic. Amphotericin B deoxycholate (cAMB), an anti-fungal compound, was shown to effectively inhibit the growth of *E. multilocularis* metacestodes, first *in vitro*, and subsequently in human patients *in vivo* [41–43]. A major limitation of cAMB is the intravenous route of administration, which makes it unsuitable for prolonged use, except for salvage treatment. Also, the effect of cAMB is only parasitostatic and the drug is nephrotoxic. Nevertheless, prolonged application of cAMB for months to years may be feasible in some cases, as side effects are mild and serious organ damage does not appear to occur.

Stadelmann et al. [55] developed and validated a novel *E. multilocularis* drug screening assay based on release of the enzyme phosphoglucose isomerase (EmPGI) by parasites that suffer from impaired viability due to drug treatment *in vitro.* The increase in PGI activity measured in culture supernatants correlated with the progressive degeneration and destruction of metacestode tissue in a time- and concentration-dependent manner. Therefore, the PGI-assay enables assessment of the degree of damage induced by drug treatment, and enables structure-activity relationship analyses. In combination with the improved large-scale *in vitro* culture techniques for metacestodes, this assay represents a highly useful and convenient tool to perform first-round *in vitro* tests on the efficacy of large numbers of anti-parasitic compounds. Anti-malarial compounds such as artemisinin and respectively derived peroxides (ozoids) [18, 28, 49], mefloquine and mefloquine enantiomers [23, 56], as well as pentamidine and dicationic pentamidine-derivatives such as diamidines, arylimidamides and diguanidino derivatives [26, 56] have been evaluated using *in vitro* cultured metacestodes. Besides albendazole, other benzimidazole derivatives have also been assessed. Of these, triclabendazole was reported to exhibit profound *in vitro* activity by Richter et al. [45], and fenbendazole, oxfendazole (fenbendazole sulfoxide) and febantel were shown to be active against *E. multilocularis* metacestodes *in vitro* and in the secondary mouse model [29]. Interestingly, TEM analysis of both albendazole and fenbendazole-treated metacestodes revealed a dramatic shortening/retraction of microtriches as a hallmark of benzimidazole action, and as a consequence separation of the acellular laminated layer from the cellular germinal layer [16, 32]. Since TEM did not reveal any microtubule-based structures within *Echinococcus* microtriches, this effect cannot be explained by the previously described mechanism of action of benzimidazoles targeting β-tubulin. Therefore, benzimidazoles must interact with additional targets that have not yet been identified [29].

In terms of *in vivo* efficacy, the anti-malarial mefloquine and the thiophene-di-guanidino compound DB1127 were shown to exhibit promising properties. Intraperitoneal application of mefloquine in secondarily-infected mice (25 mg/kg, twice a week) resulted in a reduction in parasite weight that was similar to what was obtained with orally applied albendazole (200 mg/kg/day), but oral application of the same dose was not successful [23]. However, more recent studies have now shown that the success of oral application of mefloquine in mice is dose-dependent, and at a higher dosage, results in a reduction in parasite weight comparable to what is achieved by albendazole treatment (manuscript in preparation). Similarly, intraperitoneal application of DB1127 in secondarily infected mice reduced the parasite weight, however, oral application of the drug had no effect. Although these are promising results, it is important to keep in mind that DB1127 is an experimental compound, and there is no data on bioavailability, pharmacokinetics and toxicity in animals. In addition, mefloquine treatment in humans has a variety of documented adverse side effects including neurotoxicity and elevation of liver enzymes. In conjunction with albendazole, the latter could pose a serious clinical problem. Nevertheless, inclusion of mefloquine in an albendazole-based treatment regimen at a prophylactic dosage in addition to continuous albendazole treatment might be an intriguing new concept.

The anti-echinococcal properties of the antibiotic macrolide clarithromycin were identified via an *in silico* approach [32]. Clarithromycin inhibits protein synthesis in bacteria by binding to the nascent peptide exit tunnel on the ribosome near the peptidyltransferase centre of large subunit rRNA. Higher eukaryotes carry a guanine at position 2058 of both cytoplasmic and mitochondrial rRNAs, and this modification at this position had been demonstrated to confer the resistance of eukaryotic cells to macrolide antibiotics. In contrast, the mitochondrial rRNA of *E. multilocularis* carries an adenine at sequence position 2058, which predicts susceptibility as in bacteria [46], while the nucleus-encoded rRNA is characterized by a guanine at 2058. Upon *in vitro* culture of *E. multilocularis* metacestodes with clarithromycin, parasites, as expected, exhibited severely impaired growth characteristics and morphology in a dose-dependent manner. Adult worms were also severely affected, lost their motility and displayed morphological alterations such as shortening and constriction of proglottids and increased vacuolization [32]. However, these promising results were never followed-up by appropriate *in vivo* studies.

The availability of comprehensive and detailed genome and transcriptome information on *Echinococcus* and other cestode parasites [64, 69] has opened the door for the identification of drug targets by affinity chromatography followed by MS-based sequencing, and potentially also for reverse genetics by overexpressing or silencing genes of interest. After the development of procedures to perform transient transfections of primary cells of *E. multilocularis* [52], the successful establishment of RNAi in primary *E. multilocularis* cell cultures using three *E. multilocularis* genes, *emgapdh*, *em14-3-3* and *emelp* was achieved [54]. The expression of all three genes was knocked down by RNAi on the transcriptional and translational level. In future work, this promising technology will be employed to validate drug targets or other essential proteins in the context of the immunological host-parasite interaction.

### 3.2. Drugs inhibiting cancer cell proliferation

*E. multilocularis* metacestodes and malignant tumours share some distinct features, namely their capacity for continuous cell proliferation, the potential to modulate immune response, the secretion of proteolytic enzymes in order to reach their target sites or organs, induction of angiogenesis, overexpression of 14-3-3 proteins, and the capacity of metastasis formation. These similarities suggested that anti-proliferative compounds could also affect *E. multilocularis* metacestodes. Conversely, and not surprisingly, many compounds that exhibit broad-spectrum anti-infective activities (including artemisinins, mefloquine and nitazoxanide-derivatives) also show clearly elevated toxicity in proliferating cells [22, 35].

One of the first anti-cancer drugs that had been investigated for its potential use against AE is doxorubicin, or hydroxyldaunorubicin, a DNA-interacting drug used widely in the treatment of a wide range of cancers. The compound was bound to polyisohexylcyanoacrylate nanoparticles (a colloidal biodegradable drug carrier) and applied in *E. multilocularis*-infected mice. This resulted in a reduced mass of metacestode tissue in the liver and reduced viability of this metacestode. In contrast, free doxorubicin or unbound nanoparticles had no anti-parasitic activity [30]. However, due to the massive side effects that are generally encountered by doxorubicin, this treatment approach was not further pursued.

Another class of anti-tumour agents with proven anti-parasitic activities includes isoflavonoids. Isoflavonoids are substances formed by plant tissue in response to physiological stimuli such as infectious agents. Genistein, a major component of soya, inhibits growth and metastasis formation of a number of cancer cell lines (breast, prostate, skin, colon). Genistein also stimulates the synthesis of TGF-β, which itself inhibits cancer cell proliferation [34]. Besides other targets, genistein acts on a number of signalling pathways, by functioning as a kinase inhibitor (tyrosine-kinase, MAP kinase, ribosomal S6 kinase), but also binds the oestrogen receptor-β, and thus exerts unfavourable effects on long-term treatment [39]. However, genistein, as well as a number of derivatives such as Rm6423 that do not meet the steric requirements to bind to the oestrogen receptor-β were also highly effective against *E. multilocularis* metacestodes *in vitro*, as well as against *E. granulosus s.l.* metacestodes and protoscoleces [36]. The molecular basis of the efficacy of genistein and its derivative Rm6423 has not been elucidated, but these compounds could interfere in signalling, for instance through inhibiting the tyrosine kinase activity associated with the epidermal growth factor receptor identified in *E. multilocularis* [51]. However, the anti-echinococcal efficacy of isoflavonoids has not been assessed *in vivo* to date.

An endogenous metabolite of oestrogen with both anti-angiogenic and anti-tumour effects, 2-methoxyestradiol (2-ME) was shown to induce severe damage to *E. multilocularis* metacestodes *in vitro* in a dose-dependent manner [50]. In contrast, 2-ME-treatment of experimentally infected mice did not result in a reduction in parasite weight compared to the non-treated controls. Best results were achieved with a treatment using a combination of 2-ME and albendazole, which leads to a substantial, but not statistically significant, reduction in parasite weight compared to albendazole treatment alone.

Gelmedin et al. [10] identified pyridinyl imidazoles as ATP-competitive inhibitors of a p38-like mitogen-activated protein kinase (MAPK) of *E. multilocularis* by adding them to *in vitro* cultures, and demonstrating death of parasite vesicles at concentrations that did not affect cultured mammalian cells. Imatinib, a tyrosine-kinase inhibitor used in the treatment of multiple cancers, most notably chronic myelogenous leukaemia, was reported to be highly effective in killing *Echinococcus* stem cells, metacestodes and protoscoleces *in vitro*. At concentrations as low as 10 μM, imatinib significantly inhibited the formation of metacestodes from parasite stem cells, inactivated 50% of vesicles after 7 days, and induced morphological alterations in the metacestode upon short-term treatment [11]. Another kinase inhibitor, BI 2536, a Polo-like kinase inhibitor that has been tested in clinical trials against cancer, significantly blocked the formation of metacestode vesicles from cultivated *Echinococcus* germinative cells at concentrations as low as 20 nM [62]. Furthermore, low concentrations of BI 2536 eliminated the germinative cell population from mature metacestode vesicles *in vitro*, yielding parasite tissue that was no longer capable of proliferation. As germinative cells are decisive for parasite proliferation and metastasis formation within the host, BI 2536 and related compounds are very promising compounds to complement benzimidazoles in AE chemotherapy [47].

NTZ, previously introduced as an anti-infective drug, also inhibited the proliferation of colon cancer cells *in vitro*, and Müller et al. [35] have shown that this happens by interfering with, and inhibiting, the activity of glutathione-S-transferase (GST) class π, an isoform overexpressed in many proliferating cells. In *E. granulosus s.l.* and *E. multilocularis*, the only GSTs characterized so far, have some sequence homologies to the mammalian class μ [9, 31]. The catalytic properties of recombinant GST of *E. multilocularis*, however, exhibit higher similarities to mammalian classes α and π, with, especially, a high conjugating activity on ethacrynic acid, another anti-cancer drug [31]. In principle, GSTs may have two opposite effects on drugs, namely by inactivating drugs or by activating ineffective prodrugs. Especially the latter effects have been employed as an anti-cancer drug strategy and may be further developed against AE. In addition, these studies suggest that *Echinococcus* GST should be further investigated, and validated, as a potential drug target.

Organometallic ruthenium complexes have been developed as a replacement for the highly toxic cisplatin for the treatment of cancer. Two series of η(6)-arene ruthenium(II) phosphite complexes were prepared, characterized and evaluated *in vitro* for their toxic potential against *E. multilocularis* metacestodes, with promising results [25]. Animal experimentation is, however, still pending.

More recently, screening of a commercially available FDA-approved drug library comprised of over 400 drugs that are currently in use against different diseases resulted in the identification of the profound activity of bortezomib, a proteasome inhibitor used as an anti-cancer drug, against *E. multilocularis* metacestodes. Detailed studies on the activity of bortezomib in *Echinococcus* showed that indeed the proteasome represents a promising drug target that should be exploited in more detail in the future [57].

Conversely to inhibiting metacestode proliferation, cytostatic drugs can also exhibit effects that lead to increased proliferation and growth of *E. multilocularis* metacestodes *in vivo* [15]. It was shown that *in vitro* exposure of metacestodes to methotrexate and subsequent infection of mice with treated parasites lead to massive growth and increased parasite proliferation, while navelbine and vincristine treatment had no clear effects. In this study, magnetic resonance imaging was used to monitor the effects of the drugs on growth progression [15].

## 4. Future developments

As outlined above, a number of potentially beneficial drugs and drug targets have now been identified, which could be further exploited to develop novel therapeutic treatment options. However, in order to identify compounds that will actually make it into the clinics, the currently employed tools for the identification and assessment of drug efficacy *in vitro* and *in vivo* need to be improved, both in terms of throughput as well as quality of the read-out.

In terms of *in vitro* assessment, the currently used PGI screening assay [38] has proven very valuable, but it also has some limitations: (i) the assay exhibits a certain degree of variability, depending on the age and size of metacestodes, and ideally repetitions are required with different batches of metacestode cultures to obtain clear-cut results; (ii) the assay is relatively labour-intensive and requires multiple pipetting steps; (iii) while 96 well format is possible, 24 well format is more reliable; (iv) compounds interfering with the PGI enzyme reaction could potentially disturb the assay; (v) the assay picks up metacestode damage but does not necessarily measure loss of viability; and (vi) if metacestodes are damaged by mechanisms that do not lead to PGI release within the given time frame of treatment (such as with benzimidazoles [45, 55]), false-negative results can occur. To overcome pitfalls (i)–(iv), screening assays based on *E. multilocularis* cell cultures could provide a worthwhile alternative by increasing throughput of drug screening activities. In addition, to improve pitfalls (v) and (vi), other assays that deliver a more precise read-out on metacestode viability should be developed to increase the chances of picking up drugs that hit essential targets and act as parasiticidals.

There is also ample room for improvement and refinement for the *in vivo* assessment of drug efficacy in murine AE. In an ideal world, experiments should be carried out in mice infected orally with *E. multilocularis* eggs. However, these are not always available, thus in most cases the secondary infection model (intraperitoneal injection of metacestode tissue) is used. Corresponding experiments and drug applications often take several months, and optimization of treatment parameters (stop or continuation, dosage adjustments) during this period is not possible. In addition, the classical read-out (determination of parasite weight) has a high degree of variability, due to the fact that metacestodes are often intermingled and/or encapsulated by connective tissue, or even spread to neighbouring organs, and thus the degree of encapsulation has a major impact on parasite weight. Thus, large group sizes of 8–10 animals are required. To overcome this problem, periodic ultrasound monitoring of mice under treatment could provide additional information on location, parasite mass, degree of encapsulation and organ involvement during the experimental period, and this could allow informed decisions on treatment interruption and dosage adjustments. A first step in this direction has been the development of a subcutaneous murine infection model for AE, which allowed a much more precise assessment of parasite mass, and thus drug efficacy, compared to the classical intraperitoneal infection model [27].

Another method of refinement concerns the drug application: compounds that are routinely applied by gavage on a daily basis can often be emulsified in honey, and we found that mice will take them up voluntarily, which significantly reduces the stress level during the treatment period [24]. Compared with gavage, voluntary ingestion of a drug in honey is more rapid, less stressful to the animal and less technically demanding for the administrator. As shown for albendazole, drug metabolism was not affected by this procedure, but this will have to be evaluated for other compounds [68].

## 5. Conclusions

The search for improved treatment options for echinococcosis based on novel compounds is ongoing. The significant advances in our knowledge on *Echinococcus* biology at the molecular level, and the development of reliable tools for improved *in vitro* culture techniques and genetic approaches have now facilitated the search for novel and potentially suitable chemotherapeutical tools that will provide infected patients with additional treatment options. A major concern is funding, since although compounds are readily being made available from interested parties, drug-screening efforts need to be financed. It is important to increase public awareness about the impact of AE and CE on society, in order to generate sufficient resources to actually carry out the research and studies on chemotherapeutically promising compound classes.
